# Design of a multi-epitope vaccine against cervical cancer using immunoinformatics approaches

**DOI:** 10.1038/s41598-021-91997-4

**Published:** 2021-06-11

**Authors:** Samira Sanami, Fatemeh Azadegan-Dehkordi, Mahmoud Rafieian-Kopaei, Majid Salehi, Maryam Ghasemi-Dehnoo, Mehran Mahooti, Morteza Alizadeh, Nader Bagheri

**Affiliations:** 1 Department of Medical Biotechnology, School of Advanced Technologies, Shahrekord University of Medical Sciences, Shahrekord, Iran; 2 Cellular and Molecular Research Center, Basic Health Sciences Institute, Shahrekord University of Medical Sciences, Shahrekord, Iran; 3 Medical Plants Research Center, Basic Health Sciences Institute, Shahrekord University of Medical Sciences, Shahrekord, Iran; 4 Department of Tissue Engineering, School of Medicine, Shahroud University of Medical Sciences, Shahroud, Iran; 5 Department of Biotechnology, Iranian Research Organization for Science and Technology, Tehran, Iran

**Keywords:** Computational biology and bioinformatics, Immunology

## Abstract

Cervical cancer, caused by human papillomavirus (HPV), is the fourth most common type of cancer among women worldwide. While HPV prophylactic vaccines are available, they have no therapeutic effects and do not clear up existing infections. This study aims to design a therapeutic vaccine against cervical cancer using reverse vaccinology. In this study, the E6 and E7 oncoproteins from HPV16 were chosen as the target antigens for epitope prediction. Cytotoxic T lymphocytes (CTL) and helper T lymphocytes (HTL) epitopes were predicted, and the best epitopes were selected based on antigenicity, allergenicity, and toxicity. The final vaccine construct was composed of the selected epitopes, along with the appropriate adjuvant and linkers. The multi-epitope vaccine was evaluated in terms of physicochemical properties, antigenicity, and allergenicity. The tertiary structure of the vaccine construct was predicted. Furthermore, several analyses were also carried out, including molecular docking, molecular dynamics (MD) simulation, and in silico cloning of the vaccine construct. The results showed that the final proposed vaccine could be considered an effective therapeutic vaccine for HPV; however, in vitro and in vivo experiments are required to validate the efficacy of this vaccine candidate.

## Introduction

Cervical cancer, with about 0.6 million cases and 0.3 million deaths per year, is the fourth most common type of cancer among women worldwide^1^. Human papillomavirus (HPV) is the most important cause of this disease, which is transmitted through sexual intercourse^2^. There are five main genera of HPV, alpha, beta, gamma, mu, and nu^3^. The most important HPVs are in the alpha genus, and they are classified into high-risk and low-risk groups based on the risk of oncogenic transformation^4^. The high-risk group includes types 16, 18, 31, 33, 35, 39, 45, 51, 52, 56, 58, 59, and 68^5^. Based on biological studies, HPV16 and 18 together cause approximately 70% of all cervical cancers^6^, and HPV16 is the most carcinogenic of the two^7^. HPV types 6, 11, 40, 42, 43, 44, 54, 61, 70, 72, and 81 are classified in the low-risk group^8^. HPV is a non-enveloped virus with a circular double-stranded DNA genome that is approximately 8 kb in length^9^. There are three regions in the HPV genome, an early region (E1, E2, E4, E5, E6, and E7), a late region (L1, L2), and a long control region (LCR)^10^.

The E6 and E7 oncoproteins are the major virus transforming proteins in high-risk HPV, and they play a role in cell proliferation, immortalization, and transformation in human epithelial cells^11^. The key function of the E6 protein in high-risk HPV types is to promote ubiquitin-mediated degradation of the p53 protein through its interaction with the E6-associated protein (E6AP)^12^. Moreover, p53 is a transcription factor that regulates the expression of genes involved in cell cycle arrest and apoptosis^12^. E7 binds to the retinoblastoma (Rb) protein, causing E2F to be released from the Rb-E2F complex and the cell to enter the S phase^13^. Since the E6 and E7 oncoproteins are essential for tumor progression, and they are consistently expressed in HPV-infected cells but not in healthy cells, they are ideal targets for the development of therapeutic HPV vaccines^14,15^.

**Figure 1:**
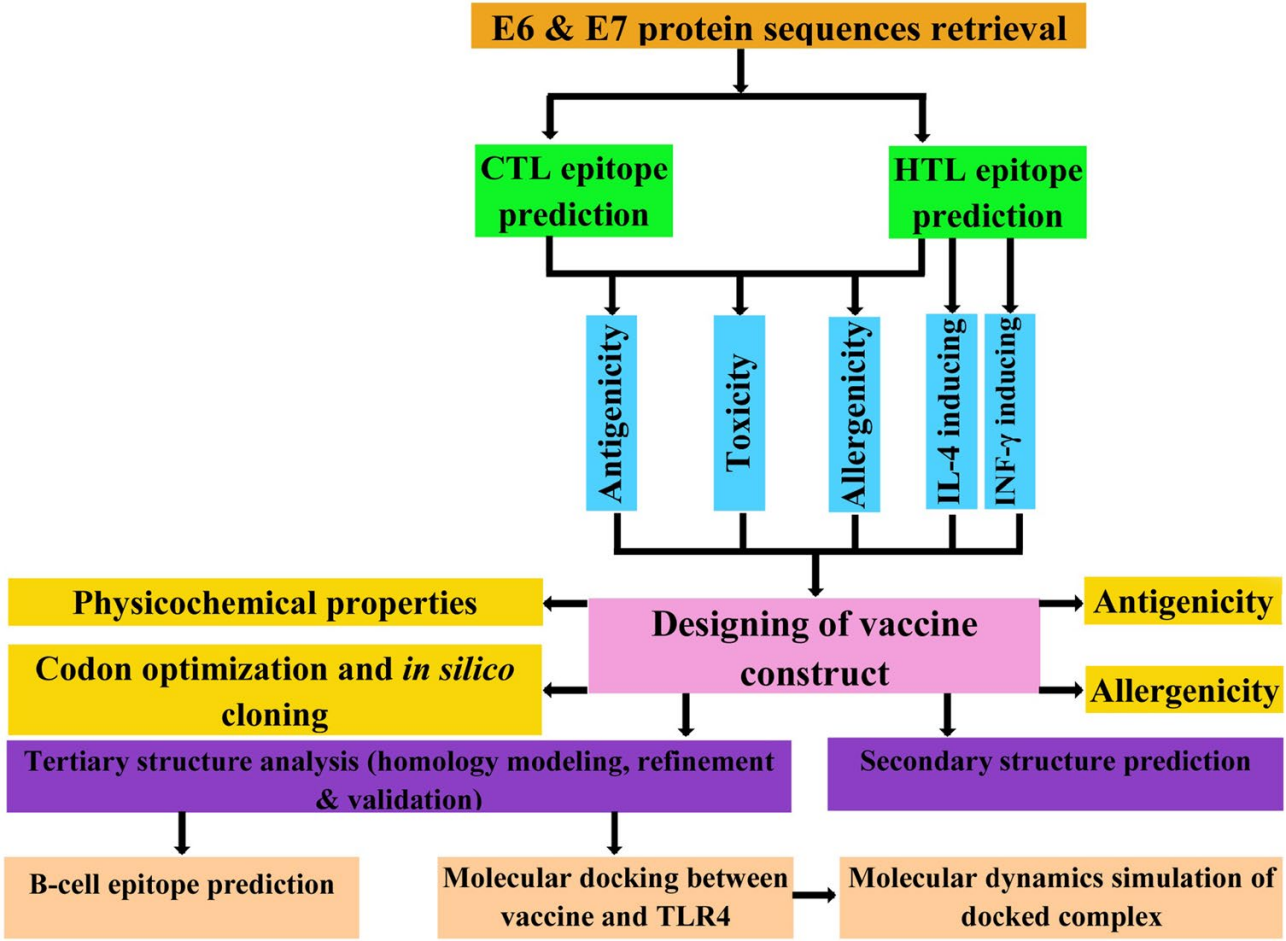
A schematic illustration of the immunoinformatics approaches used to design a multi-epitope vaccine.

Gardasil and Cervarix are available prophylactic vaccines to prevent HPV infection. These vaccines have no therapeutic effect because their action mechanism is to induce the production of neutralizing antibodies against the L1 capsid protein, and since L1 is expressed in the granular epithelium before viral shedding, consequently, current prophylactic vaccines are not effective in eliminating previous infections^16^. Inducing cell-mediated immune response is needed to clear infected cells^17^. This study aims to design a therapeutic vaccine against cervical cancer using reverse vaccinology. The reverse vaccinology method, which examines the genomes of pathogenic microorganisms to identify antigens, employs a number of algorithms for predicting T-cell and B-cell epitopes. Unlike the conventional vaccinology method, this method does not require culturing pathogens and extracting antigenic proteins, which are costly and time-consuming processes^18^. In the present study, CTL and HTL epitopes of the E6 and E7 oncoproteins were identified and linked together by appropriate linkers for the design of a multi-epitope vaccine against cervical cancer. Multi-epitope vaccines are recombinant vaccines that are considered to be a promising strategy against tumors and viral infections due to their high specificity, safety, and stability, and low-cost development^19^. The major downside of multi-epitope vaccines is their low immunogenicity because proteinases can quickly degrade the antigenic peptides in the body, making them difficult to identify by the immune cells’ receptors^20^. One of the strategies suggested for improving the immune response generated by multi-epitope vaccines is to use adjuvants in the vaccine construct^21^. We also added the 50S ribosomal protein L7/L12 (Locus RL7_MYCTU) as an adjuvant to the N-terminal of the vaccine construct with the help of an EAAAK linker. Subsequently, the physicochemical properties and the secondary and tertiary structures of the vaccine were predicted. Furthermore, several analyses were also carried out, including molecular docking, MD simulation, and in silico cloning of the vaccine construct. The flow of methods used to design a multi-epitope vaccine is illustrated in Fig. 1.

## Results

### Identification and selection of T-cell epitopes

A total of 54 CTL epitopes for the E6 and E7 proteins were predicted using the NetCTL 1.2 server. The predicted epitopes were screened at several stages. In the first step, those epitopes were selected that could bind to at least three MHC class I supertypes. These epitopes were then evaluated for antigenicity, toxicity, and allergenicity using VaxiJen v2.0, ToxinPred, and AllerTOP v. 2.0 servers, respectively. Finally, a CTL epitope was selected for each of the E6 and E7 proteins (Table 1). Here, we predicted 99 HTL epitopes for the E6 and E7 proteins using the NetMHCII 2.3 server, among which 27 epitopes capable of binding to at least three MHC class II alleles were identified and checked for antigenicity, toxicity, and allergenicity. The selection of six HTL epitopes for E6 and one epitope for E7 was the result of these screenings (Table 2).

**Table 1:** Predicted CTL epitopes of E6 and E7 proteins.

Protein	CTL epitope	MHC class I supertypes	VaxiJen score	Allergenicity	Toxicity	Final decision
E6	IVYRDGNPY	A26, A3, B62	0.248	Allergen	Non-toxin	–
	MHQKRTAMF	A24, B39, B8	1.2253	Allergen	Non-toxin	–
	RREVYDFAF	B8, B27, B39	1.3879	Non-allergen	Non-toxin	*
E7	RAHYNIVTF	A24, B7,B8, B58,B62	0.5919	Non-allergen	Non-toxin	*

**Table 2:** Predicted HTL epitopes of E6 and E7 proteins.

Protein	HTL epitope	MHC class II alleles	VaxiJen score	Allergenicity	Toxicity	IFN-γ –inducing	IL-4- inducing	Final decision
E6	YRHYCYSLYGTTLEQ	DRB1_0101,DRB1_0901, DRB1_0405	0.8524	Non-allergen	Non-toxin	Positive	IL4-inducer	*
	CIVYRDGNPYAVCDK	DRB1_0401, DRB1_0301, DRB3_0101, DRB3_0202, DRB3_0202	0.43	Allergen	Non-toxin	Positive	IL4-inducer	–
	CKQQLLRREVYDFAF	DRB1_0103, DRB3_0101, DRB4_0101, DRB4_0103	0.051	Allergen	Non-toxin	Positive	IL4-inducer	–
	DKKQRFHNIRGRWTG	DRB1_0103, DRB1_1301, DRB4_0103, DRB5_0101	0.7454	Allergen	Non-toxin	Positive	IL4-inducer	-
	DLCIVYRDGNPYAVC	DRB1_0401, DRB1_1302, DRB3_0101, DRB3_0202, DRB1_0301	0.8357	Allergen	Non-toxin	Positive	IL4-inducer	–
	KFYSKISEYRHYCYS	DRB1_1501, DRB1_1602, DRB5_0101	0.6924	Allergen	Non-toxin	Positive	IL4-inducer	–
	KKQRFHNIRGRWTGR	DRB1_0103, DRB1_0801, DRB1_1301, DRB4_0103, DRB5_0101	1.0979	Allergen	Non-toxin	Positive	IL4-inducer	–
	KQQLLRREVYDFAFR	DRB3_0101, DRB4_0101, DRB4_0103	0.2691	Allergen	Non-toxin	Positive	IL4-inducer	–
	KQRFHNIRGRWTGRC	DRB1_0103, DRB1_0103, DRB4_0103, DRB5_0101, DRB1_0801	1.347	Allergen	Non-toxin	Positive	IL4-inducer	–
	LKFYSKISEYRHYCY	DRB1_1501, DRB1_1602, DRB5_0101, DRB1_0801	0.8558	Allergen	Non-toxin	Positive	IL4-inducer	–
	QQLLRREVYDFAFRD	DRB1_0301, DRB3_0101, DRB4_0101	0.5512	Allergen	Non-toxin	Positive	Non-IL4-inducer	–
	QRFHNIRGRWTGRCM	DRB1_1301, DRB4_0103, DRB1_0103, DRB1_0801	1.3041	Allergen	Non-toxin	Positive	IL4-inducer	–
	RDLCIVYRDGNPYAV	DRB1_0401, DRB1_0301, DRB1_1302, DRB3_0101	0.9706	Allergen	Non-toxin	Positive	IL4-inducer	–
	CDKCLKFYSKISEYR	DRB1_1602, DRB1_0801, DRB1_1501, DRB1_0802	0.2837	Non-allergen	Non-toxin	Positive	IL4-inducer	–
	CLKFYSKISEYRHYC	DRB1_1602, DRB1_0801, DRB1_0802 , DRB1_1501, DRB5_0101	0.7518	Non-allergen	Non-toxin	Positive	IL4-inducer	*
	DKCLKFYSKISEYRH	DRB1_1602, DRB1_0801, DRB1_1501, DRB5_0101, DRB1_0802	0.26	Non-allergen	Non-toxin	Positive	IL4-inducer	–
	EYRHYCYSLYGTTLE	DRB1_0101, DRB1_0405, DRB1_0901	1.1925	Non-allergen	Toxin	Positive	IL4-inducer	–
	HLDKKQRFHNIRGRW	DRB1_0103, DRB4_0103, DRB1_1301	0.7193	Non-allergen	Non-toxin	Positive	IL4-inducer	*
	KCLKFYSKISEYRHY	DRB1_1602, DRB1_0802, DRB1_0802 , DRB1_1501, DRB5_0101,DRB1_1101	0.4568	Non-allergen	Non-toxin	Positive	IL4-inducer	*
	LCIVYRDGNPYAVCD	DRB1_0301, DRB1_0401, DRB3_0101, DRB3_0202, DRB3_0202	0.6622	Non-allergen	Non-toxin	Positive	IL4-inducer	*
	LDKKQRFHNIRGRWT	DRB4_0103, DRB1_0103, DRB1_1301	0.9320	Non-allergen	Non-toxin	Positive	IL4-inducer	*
	VYCKQQLLRREVYDF	DRB1_0103, DRB1_1301, DRB4_0103	0.0419	Non-allergen	Non-toxin	Positive	IL4-inducer	–
E7	LRLCVQSTHVDIRTL	DRB1_0301, DRB1_0701, DRB1_0801, DRB4_0101, DRB1_0301, DRB1_0403	0.7711	Allergen	Non-toxin	Positive	IL4-inducer	–
	STLRLCVQSTHVDIR	DRB1_0403, DRB1_0404, DRB1_0701, DRB1_0801, DRB3_0301, DRB4_0101,DRB4_0103	0.8539	Allergen	Non-toxin	Positive	IL4-inducer	–
	TLRLCVQSTHVDIRT	DRB1_0701, DRB1_0801, DRB3_0301, DRB4_0101,DRB4_0103	0.7219	Allergen	Non-toxin	Positive	IL4-inducer	–
	DSTLRLCVQSTHVDI	DRB1_0403, DRB1_0404,DRB1_0701,DRB1_0801 , DRB3_0301, DRB4_0101	0.5514	Non-allergen	Non-toxin	Positive	IL4-inducer	*
	IVTFCCKCDSTLRLC	DRB1_0301, DRB1_0403, DRB3_0101	0.3626	Non-allergen	Toxin	Positive	Non-IL4-inducer	–

### Construction of the multi-epitope vaccine construct

**Figure 2:**
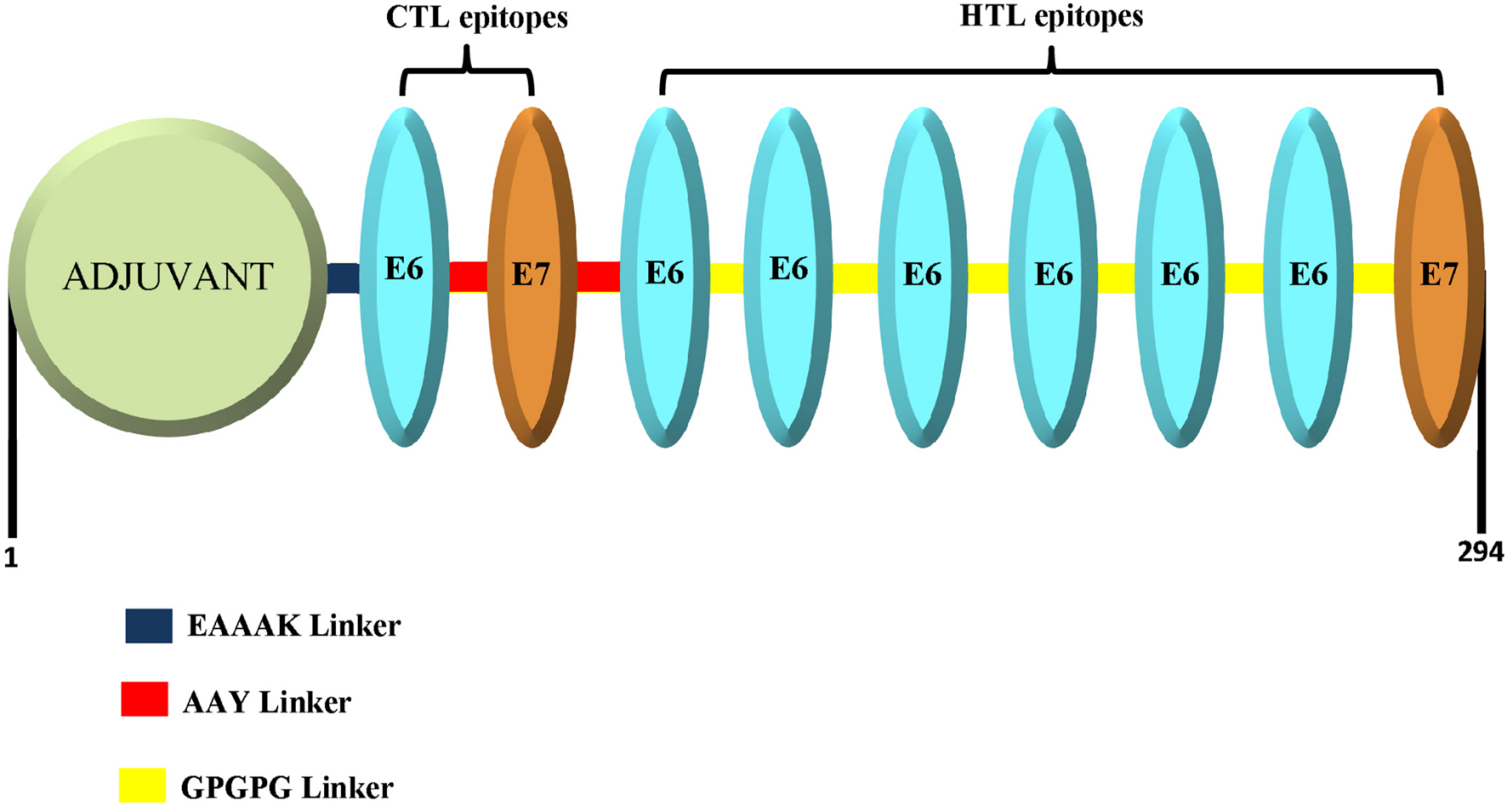
The structural arrangement of the final multi-epitope vaccine construct.

A total of two CTL epitopes and seven HTL epitopes were merged to construct the multi-epitope vaccine using AAY and GPGPG linkers, respectively. A sequence of 159 amino acids was generated after epitope fusion. The adjuvant sequence, with a length of 130 amino acid (MAKLSTDELLDAFKEMTLLELSDFVKKFEETFEVTAAAPVAVAAAGAAPAGAAVEAAEEQSEFDVILEAAGDKKIGVIKVVREIVSGLGLKEAKDLVDGAPKPLLEKVAKEAADEAKAKLEAAGATVTVK), was added to the N-terminal of the vaccine sequence by an EAAAK linker. The final designed vaccine construct consisted of 294 amino acids (Fig. 2).

### Evaluation of the antigenicity, allergenicity, and physicochemical properties of the vaccine

The antigenicity of the vaccine construct was calculated using the VaxiJen v2.0 and ANTIGENpro servers. The probability of antigenicity predicted by VaxiJen v2.0 and ANTIGENpro was 0.5058 and 0.745186, respectively. The allergenicity of the proposed vaccine was predicted using the AllerTOP v. 2.0 server, indicating that it was non-allergenic. Various physicochemical characteristics of the designed vaccine were calculated using the ProtParam server. The final composition of the multi-epitope vaccine consists of 294 amino acids. The theoretical pI, molecular weight, and instability index of the vaccine construct were calculated to be 8.33, 32.01 kDa, and 37.82, respectively. The half-life of the vaccine was estimated to be 30 h in mammalian reticulocytes, more than 20 h in yeast, and more than 10 h in *E. coli*. The aliphatic index of the vaccine was 76.43, and its GRAVY score was reported to be -0.307.

### Prediction of the secondary structure

**Figure 3:**
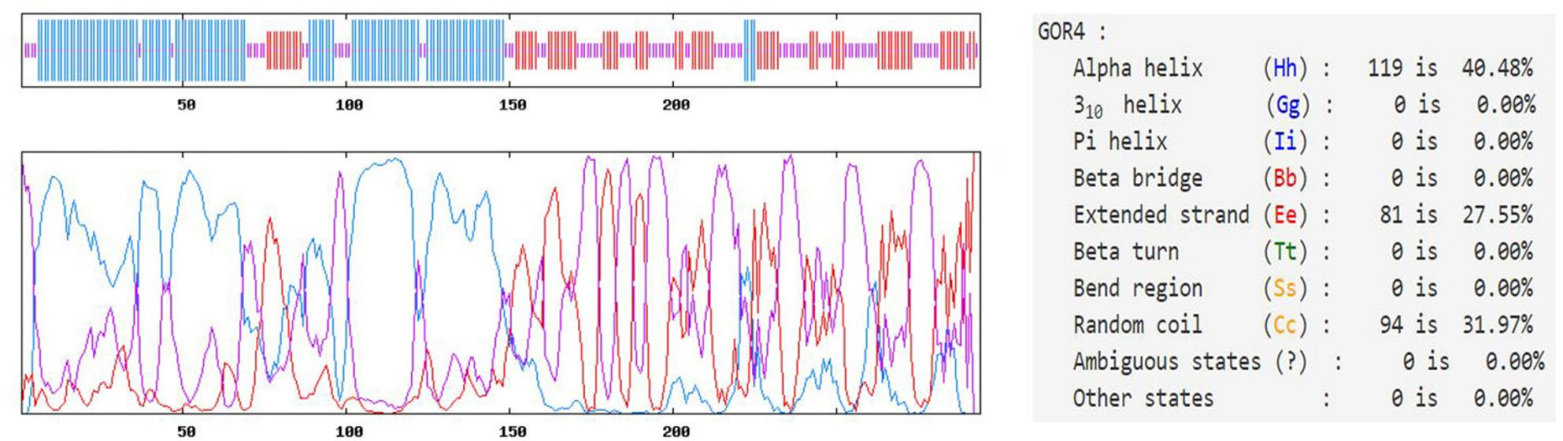
The graphical representation of the secondary structure configuration of the multi-epitope vaccine.

The percentage of the secondary structure components of the multi-epitope vaccine was computed using the Prabi server. The predicted structure included alpha-helix (40.48%), extended strand (27.55%), and random coil (31.97%) (Fig. 3).

### Tertiary structure modeling, refinement, and validation of the multi-epitope vaccine

**Figure 4:**
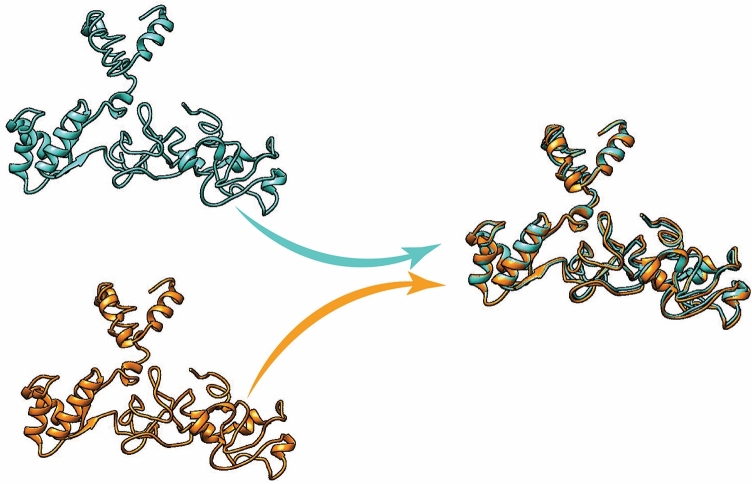
The unrefined and refined structures of the 3D model of the vaccine. The unrefined structure is shown in blue, while the refined structure is shown in orange. To identify the differences between the unrefined and refined structures, the structures were superimposed.

**Figure 5:**
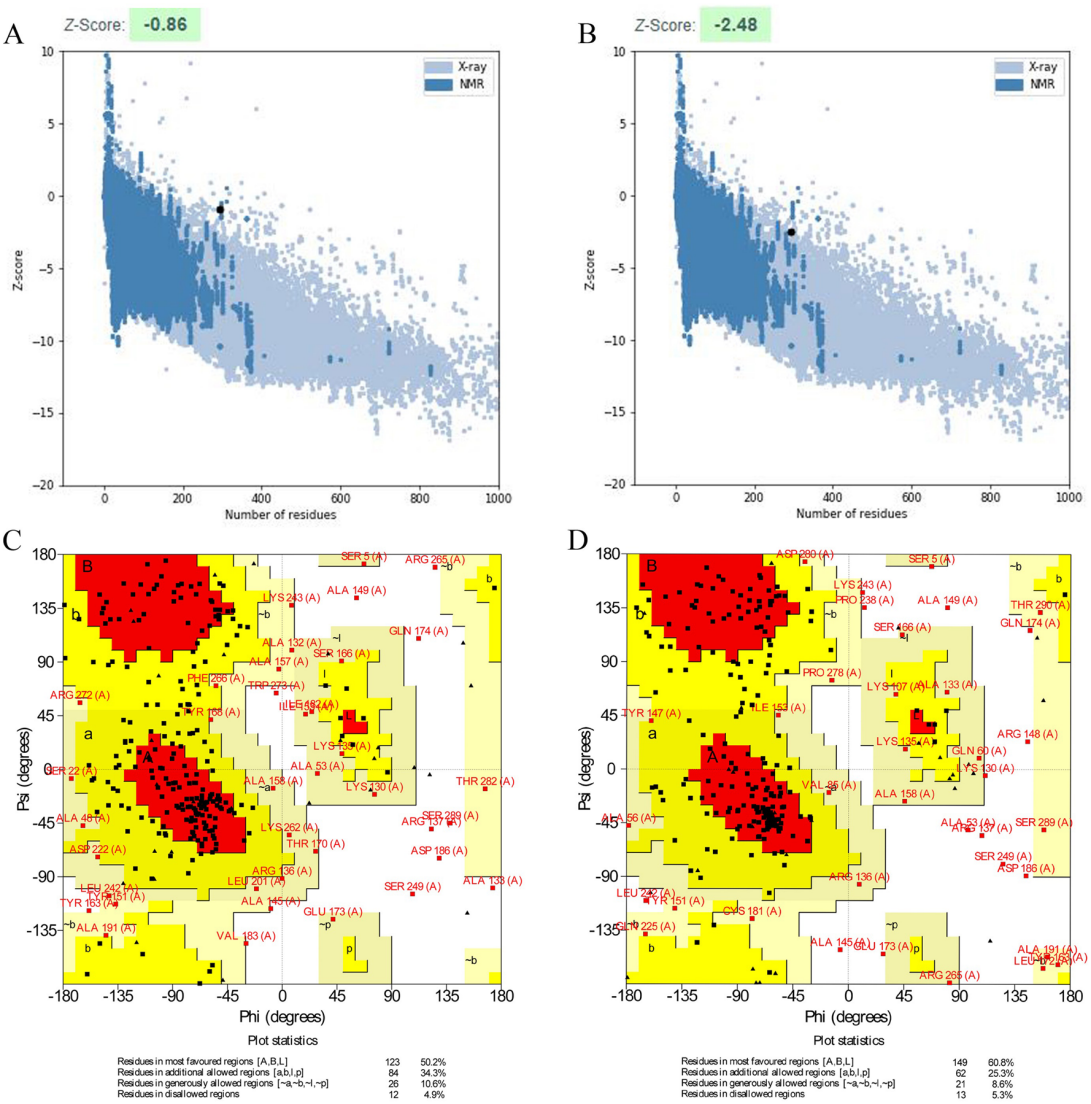
Evaluation of the 3D model of the vaccine construct using the ProSA-web and Ramachandran plot before and after the refining. (**A**) The initial model has a Z score of − 0.86, (**B**) while the refined model has a Z score of − 2.48. (**C**) The Ramachandran plot analysis shows that in the initial model, 50.2%, 34.3%, 10.6%, and 4.9% of the residues are found in the favoured, additional allowed, generously allowed, and disallowed regions, respectively, (**D**) while in the refined model, these values changed to 60.8%, 25.3%, 8.6% and 5.3%, respectively.

The five models of the 3D structure of the vaccine construct were generated by the I-TASSER server using the threading templates (PDB Hit: 1dd4A, 6slmA, 1rquA, 4giz, 1dd3A, 4gizC, 2ftc, 1dd4A, and 6slm). The calculated C-score values for models 1–5 were − 3.72, − 3.94, − 4.10, − 4.43, and − 4.44, respectively. The C-score is usually within the range of − 5 to 2, where a higher C-score for the model indicates that it has a high level of confidence^22^. Therefore, we selected model 1 with a C-score value of − 3.72. Chimera 1.15rc software was used to visualize the 3D model of the vaccine construct^23^ (Fig. 4). This model was then refined by the 3Drefine server. This server provided five refined models with different parameters, including the 3D refined score, GDT-TS, GDTHA, RMSD, MolProbity, and RWPlus (Table 3). Higher GDT-TS, GDT-HA, and RMSD values, and lower 3D refine Score, RWplus, and MolProbity values indicate a higher quality for the models. The refined model 5 was selected based on the above parameters (Fig. 4). The ProSA-web and SAVES v6.0 servers were also used to compare the overall quality of the protein structure of the multi-epitope vaccine before and after the refining process. The Z-score of the initial and refined models was − 0.86 and − 2.48, respectively (Fig. 5A,B). The Ramachandran plot generated by the SAVES v6.0 server showed that in the initial model, 50.2%, 34.3%, 10.6%, and 4.9% of the residues were present in the favoured, additional allowed, generously-allowed, and disallowed regions, respectively Fig. 5C), while in the refined model, these values changed to 60.8%, 25.3%, 8.6% and 5.3%, respectively (Fig. 5D).

**Table 3:** Results of the model refinement.

Model	3D^refine^ Score	GDT-TS	GDT-HA	RMSD (Å)	MolProbity	RWPlus
5	26463.3	0.9456	0.8019	0.825	4.381	− 49,065.048671
4	27133.7	0.9583	0.8248	0.769	4.385	− 49,004.222934
3	28245.9	0.9711	0.8495	0.699	4.431	− 48,951.722387
2	30776.4	0.9813	0.8818	0.600	4.431	− 48,782.336975
1	37559.3	0.9966	0.9405	0.442	4.495	− 48,486.394142

Models with higher GDT-TS, GDT-HA, and RMSD values and lower 3Drefine Score, RWplus, and MolProbity values are of higher quality.

### Prediction of the B-cell epitope

**Figure 6:**
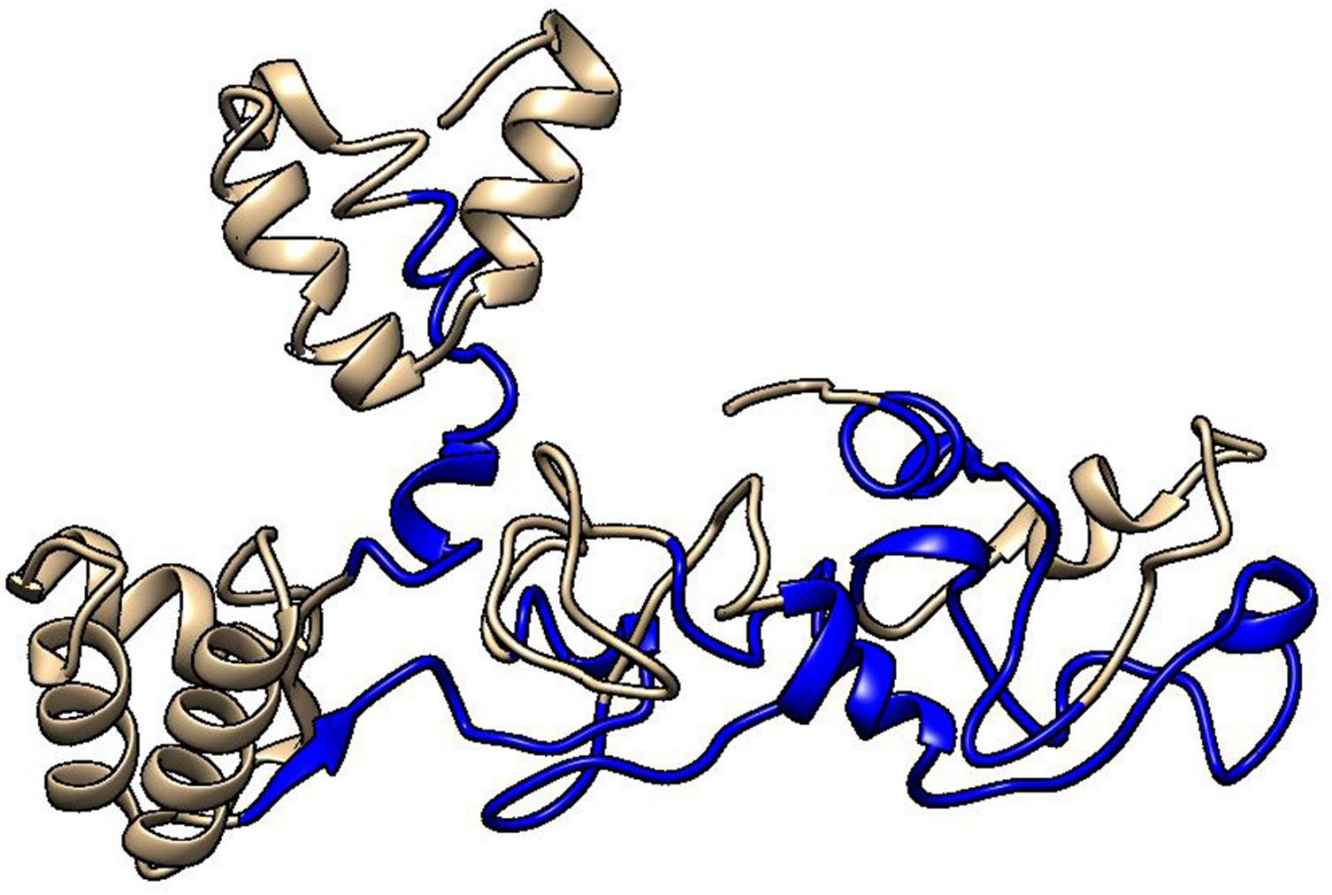
The linear B-cell epitopes (blue color) are highlighted in the 3D structure of the multi-epitope vaccine (tan color).

**Figure 7:**
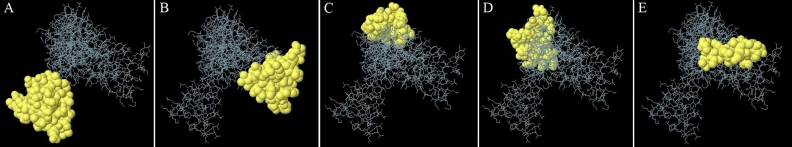
To visualize discontinuous epitopes on the vaccine construct's 3D structure (**A**–**E**), the open- source molecular viewer Jmol (http://jmol.sourceforge.net/) was employed. The gray sticks and the yellow surface indicate the vaccine construct and discontinuous B-cell epitopes, respectively.

Seven linear B-cell epitopes (20-mer) were predicted by the BCPREDS, and the scores of the epitopes ranged from 0.819 to 1 (Table 4). The position of the linear B-cell in the final vaccine construct were highlighted using the Chimera 1.15rc software^23^ (Fig. 6). The ElliPro server also predicted five discontinuous B-cell epitopes in the tertiary structure of the vaccine (Fig. 7). The minimum and maximum scores for the predicted discontinuous B-cell epitopes were 0.648 and 0.792, respectively (Table 5).

**Table 4:** A list of linear B-cell epitopes predicted by the BCPREDS.

Position	Linear B-cell epitope	Score
269	IRGRWTGPGPGDSTLRLCVQ	1
170	TTLEQGPGPGLCIVYRDGNP	1
223	KKQRFHNIRGRWGPGPGKCL	1
247	KISEYRHYGPGPGLDKKQRF	1
43	AAAGAAPAGAAVEAAEEQSE	0.999
201	LKFYSKISEYRHYCGPGPGH	0.998
126	TVTVKEAAAKRREVYDFAFA	0.819

**Table 5:** A list of discontinuous B-cell epitopes predicted by the ElliPro server.

No	Position	Discontinuous B-cell epitope	Score
1	1	MAKLSTDELLDAFKEMTLLELSDFVKKFEETFEVTAAAPVAVAAA	0.792
2	67	LEAAGDKKIGVIKVVREIVSGLGLKEAKDLVDGAP	0.744
3	209	EYRHYCGPGPGHLDKKQRFHNIRGRW	0.734
4	246	SKISEYRHYGPGPGLDKKQRFHNIRG	0.654
5	109	AKEAADEAKAKLEAAGATV	0.648

### Molecular docking

**Figure 8:**
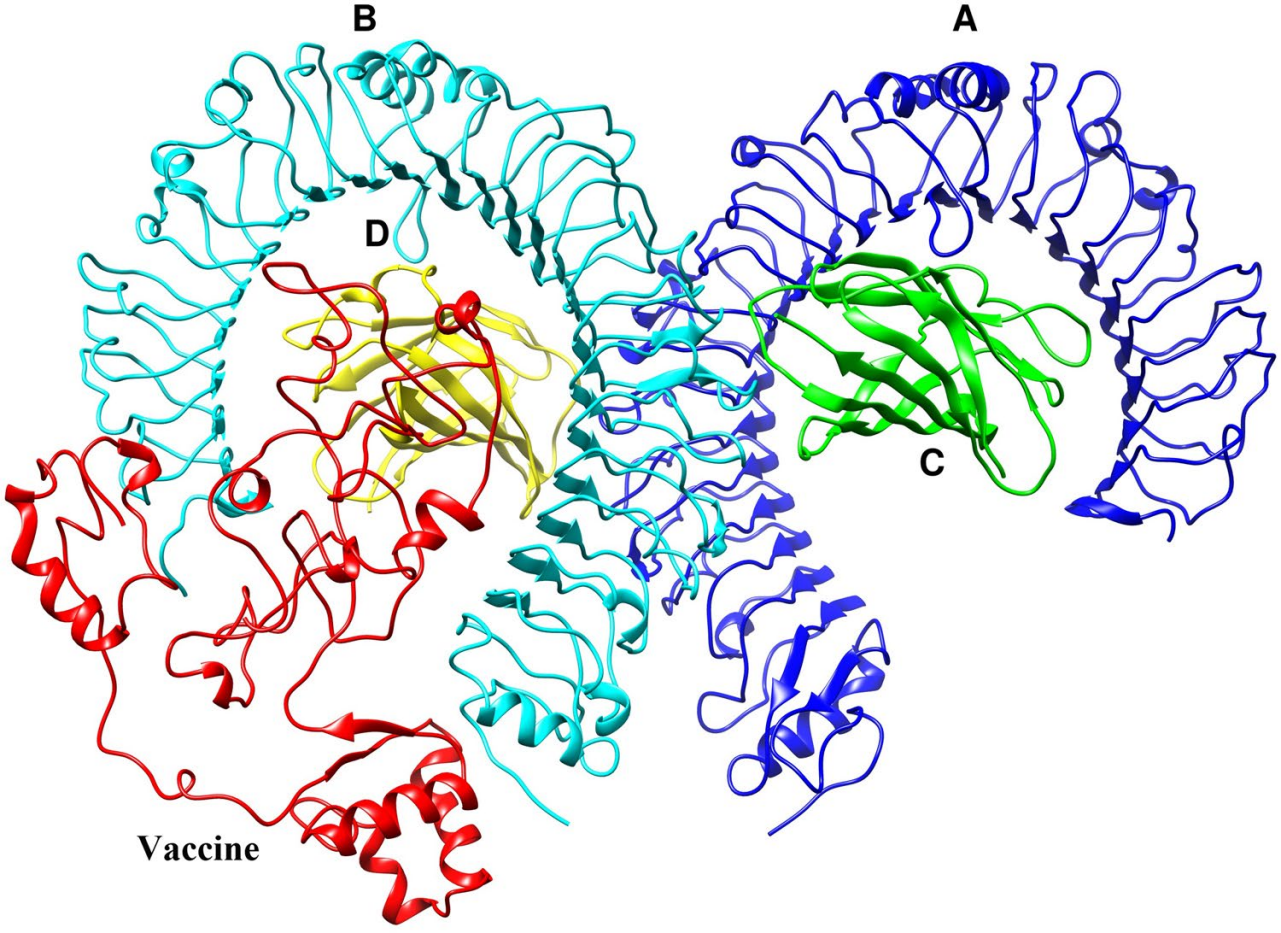
Docked complex of the vaccine construct (ligand) and TLR4 (receptor). Chains A, B, C, and D of TLR4 are shown in blue, cyan, green, and yellow, respectively, while the vaccine construct is shown in red.

**Figure 9:**
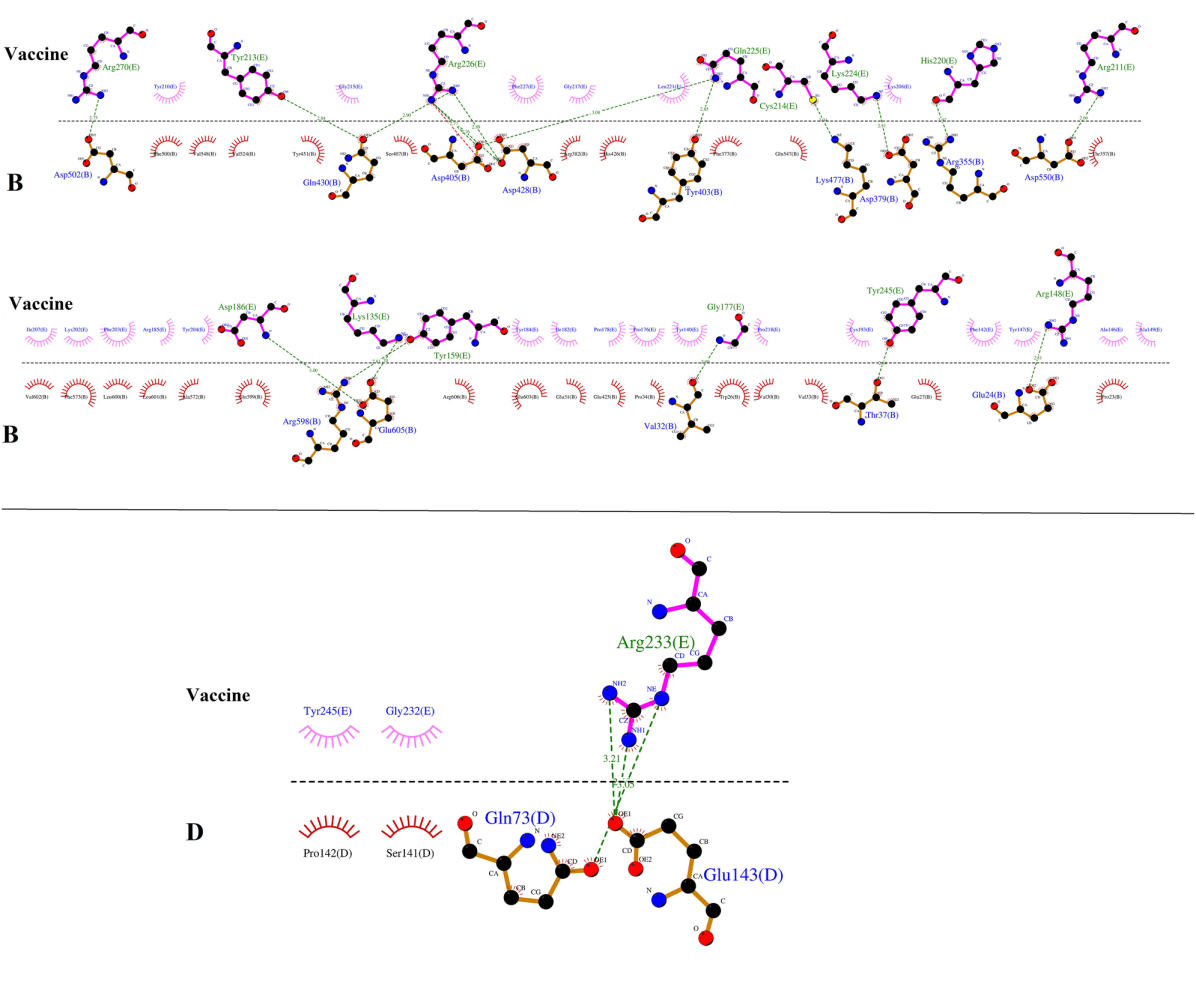
The map of interactions between the vaccine construct and TLR4.

The molecular docking between the vaccine construct and TLR4 was conducted using the ClusPro 2.0 server. In this study, the server generated 26 clusters, and it then ranked them by energy level. The cluster with the lowest energy of -1103.8 was chosen as the best complex. The Chimera 1.15rc software was used to visualize the molecular docking results^23^ (Fig. 8). There were 17 hydrogen bonds between chain B of TLR4 and the vaccine, while three hydrogen bonds formed between chain D and the vaccine. The map of hydrogen bonds and hydrophobic contacts between the vaccine construct and TLR4 generated by the LigPlot v1.4.5 program^24^ (Fig. 9). Tables 6 and 7 show the amino acids involved in the formation of these hydrogen bonds along with the lengths of the bonds.

**Table 6:** List of amino acids involved in hydrogen bonds between vaccine and TLR4 (chain B).

TLR4 (chain B)	Vaccine	Bond length (Å)
Glu605	Asp186	3
	Lys135	2.7
Arg598	Tyr159	2.81
Val32	Gly177	2.99
Thr37	Tyr245	2.84
Glu24	Arg148	2.81
Asp502	Arg270	2.75
Gln430	Tyr213	2.84
	Arg226	2.9
Asp428	Arg226	2.89
		2.76
Asp405	Arg226	2.77
	Gln225	3.08
Tyr403	Gln225	2.85
Lys477	Cys214	3.04
Asp379	Lys224	2.81
Arg225	His220	2.97
Asp550	Arg211	2.66

**Table 7:** List of amino acids involved in hydrogen bonds between vaccine and TLR4 (chain D).

TLR4 (chain D)	Vaccine	Bond length (Å)
Glu143	Arg233	3.21
		2.8
Gln73	Arg233	3.05

### MD simulation

**Figure 10:**
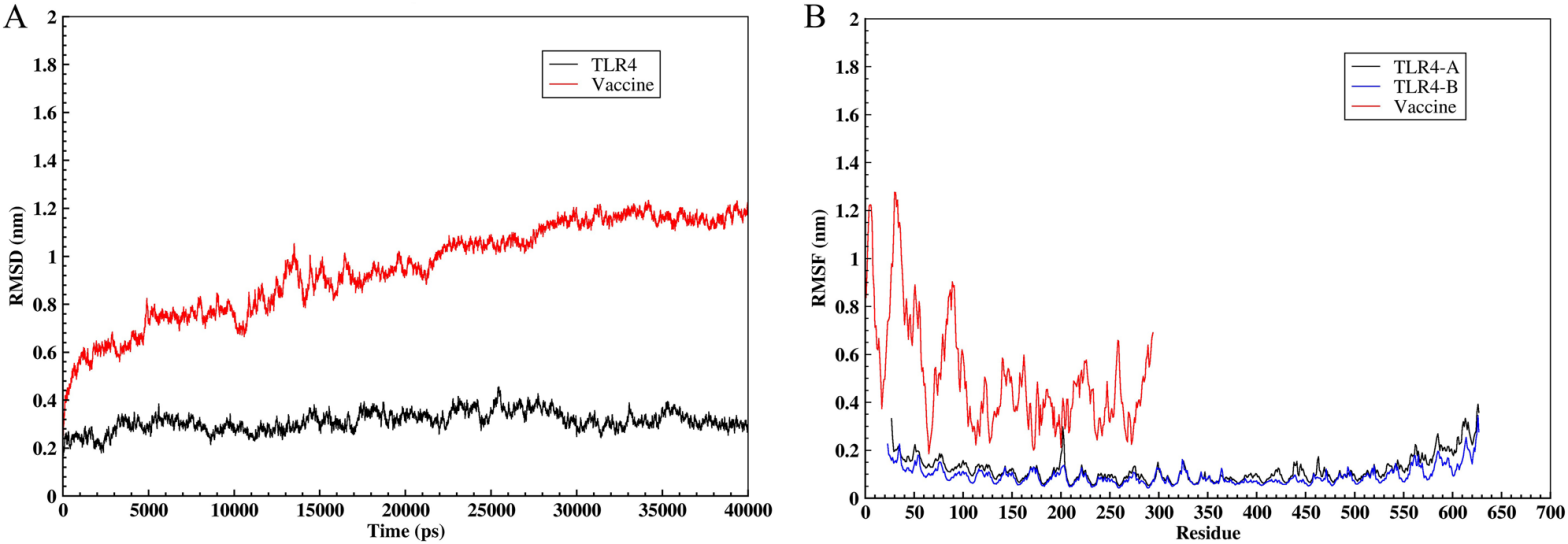
Molecular dynamics simulation of the vaccine—TLR4 complex. (**A**) RMSD plot of the vaccine-TLR4 complex. (**B**) RMSF plot of the vaccine—TLR4.

The docked complex of the vaccine construct and TLR4 was subjected to MD simulation using the GROMACS 2019.6 software. RMSD was evaluated to determine the structural stability of the vaccine and TLR4, while RMSF was calculated to measure residual fluctuations. At the beginning of the simulation, the RMSD value of TLR4 increased rapidly, reaching approximately 0.3 nm in 3000 ps, and it then fluctuated slightly around this value until the end of the simulation. The RMSD value of the vaccine had an upward trend, reaching 0.6 nm at 1000 ps. Afterward, RMSD increased slowly until it reached approximately 1.1 nm at 30,000 ps, and it remained constant at this value until the end of the simulation (Fig. 10A). In the previous section, it was observed that the vaccine construct was attached to chain B from TLR4, and since both chains A and B from TLR4 have the same sequence, the RMSF values of the two chains were compared to accurately evaluate the effects of the vaccine construct binding on the flexibility of chain B. The RMSF value of residues 30–110, 200–205, 390–470, and 550–627 from chain A showed a greater degree of flexibility than that of chain B, while the flexibility of other regions in the two chains was almost the same. The RMSF plot of the vaccine showed that most of the residues were highly flexible (Fig. 10B).

### Codon optimization and in silico cloning of the final vaccine construct

**Figure 11:**
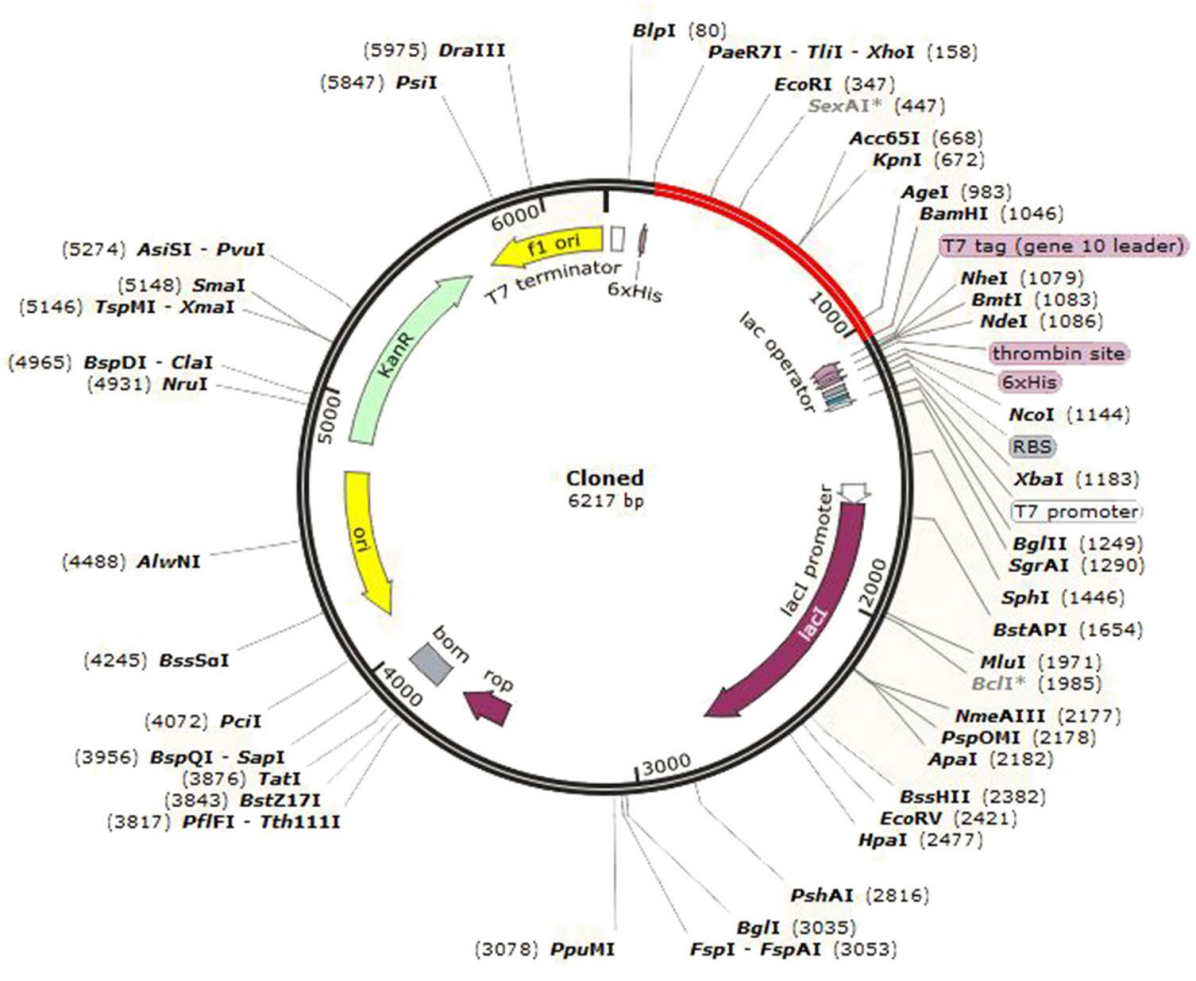
In silico cloning of the multi-epitope vaccine into the pET28a (+) vector using SnapGene sofware free-trial (https://www.snapgene.com/free-trial/). The red section represents the vaccine construct and the black section shows the backbone of the vector.

The back translation and codon optimization of the multi-epitope vaccine were performed by JCat. The CAI and GC contents of the optimized nucleotide sequence of the vaccine were 0.95 and 52.04%, respectively. Finally, in silico cloning of the vaccine construct into the pET-28 (+) vector was performed using the SnapGene software (Fig. 11).

## Discussion

Cervical cancer, caused by HPV, is a public health crisis in both developing and developed countries^25^. Despite the availability of HPV prophylactic vaccines, developing a therapeutic vaccine for cervical cancer remains an essential need in public health^26^. Reverse vaccinology, an unconventional approach to the development of new vaccines that combines immunogenicity, immunogenicity, and bioinformatics, has attracted the attention of many researchers around the world^27^. This approach has been widely used to develop multi-epitope vaccines against a variety of organisms, including *Helicobacter pylori*^28^, *Leishmania donovani*^29^, *Klebsiella pneumoniae*^30^, hepatitis C virus^31^, *Fasciola gigantica*^32^, *Tropheryma whipplei*^33^, *Elizabethkingia anopheles*^34^, *Candida auris*^35^, dengue virus^36^, Zika virus^37^, and SARS-COV-2^38–40^. In recent years, several studies have been published focusing on the design of multi-epitope vaccines for HPV. In the studies conducted by Negahdaripour et al., the HPV16 L2 protein was used to predict the epitope^41,42^, while Sarkar et al.^43^. and Mahmoudvand et al.^44^ used L1 protein to predict the epitope. In another study, Namvar et al. selected the E5 and E7 proteins from HPV16/18/31/45 as target proteins for epitope prediction^45^. The E6 and E7 proteins are key targets in the development of therapeutic vaccines against cervical cancer^46^ for a variety of reasons. First, HPV-associated malignancies and HPV-infected cells consistently express E6 and E7, whereas healthy cells do not^14,15^. Second, E6 and E7 proteins are essential for the initiation and maintenance of HPV-associated malignancies, cancer progression, and escape from the immune system^47^. Third, E6 and E7 proteins are viral antigens that are not subject to central tolerance by human immune systems^48^. Therefore, in the present study, the E6 and E7 oncoproteins from HPV16 were chosen as the target antigens for epitope prediction. The predicted epitopes were evaluated for antigenicity, toxicity, and allergenicity. In multi-epitope vaccines, the nature of the epitopes, adjuvants, and linkers, and their order and position in the chimeric sequence are all important. Since the physicochemical properties and the secondary and tertiary structures of the multi-epitope vaccine are determined by the construct’s structure, the nature of the epitopes, adjuvant, and linkers, and their arrangement and position in the multi-epitope vaccine are all important^42^. In this study, we used AAY linkers to fuse CTL epitopes, while we used GPGPG linkers to link HTL epitopes, similar to the studies by Khatoon et al.^29^, Tahir ul Qamar et al.^49^, and Tarang et al.^50^. GPGPG and AAY linkers promote epitope presentation, while they also reduce the formation of junctional epitopes^51,52^. The 50S ribosomal protein L7/L12 (Locus RL7_MYCTU) is a protein derived from *Mycobacterium tuberculosis* that has been shown in several studies to have an affinity for TLR4^53–55^; hence, we used it as an adjuvant to improve the vaccine’s immunogenicity. The EAAAK linker decreases the connection with other protein areas, while increasing stability^56,57^.

The proposed vaccine construct was antigenic and non-allergic, indicating its effectiveness in eliciting robust immune responses without causing potentially-harmful allergic responses. The theoretical pI of the vaccine was found to be 8.33, indicating that the vaccine is basic in nature. The molecular weight of the vaccine was 32.01 kDa, which is appropriate since proteins with molecular weights less than 110 kDa are easier and quicker to purify^58^. The instability index of the vaccine was calculated to be 37.82, and as this value is below 40, the vaccine is considered a stable protein^59^. The half-life of our vaccine was determined to be 30 h in mammalian reticulocytes, while the half-life of the constructs designed in the study of Sarkar et al. is one hour^43^, indicating that our vaccine is exposed to the immune system for a longer period of time than the vaccines designed by Sarkar et al. The aliphatic index of the vaccine was calculated to be 76.43, which shows that it is thermostable^60^. The GRAVY value was -0.307, and a negative value for this parameter indicates that the vaccine is hydrophilic, it can interact with water molecules^61^. However, in the study of Negahdaripour et al*.*^41^*.* GRAVY was determined to be 0.252, and the use of micelles to increase vaccine interaction inside the polar environment of the body seems to be needed due to the vaccine's hydrophobic nature.

After building the vaccine’s three-dimensional structure, the refining process was used to improve its quality, getting it closer to the native structure. Model validation is necessary to compare the quality of the unrefined model with that of the refined model. The Ramachandran plot showed that 50.2% of the residues in the unrefined model were found in the favoured region, while 60.8% of the residues in the refined model were located in the favoured region, indicating the refined model’s improvement. The TLR4 immune receptor is expressed in human cervical cancer HeLa cells with a frequency 100 times higher than other TLRs, proving a correlation between TLR4 and cervical cancer progression^62,63^. Therefore, the molecular docking analysis of the vaccine was carried out with TLR4. The molecular docking results indicated that the vaccine interacted strongly with TLR4. The vaccine-TLR4 docked complex was also subjected to MD simulation to determine the vaccine construct’s stability. The RSMD plot of the proposed vaccine and TLR4 revealed that both were stable. According to the RMSF analysis, the vaccine construct had the lowest fluctuations in the regions with the most interactions with TLR4. Codon optimization was carried out in order to increase the expression of the vaccine candidate in *E. coli* (K12 strain). The vaccine sequence had a CAI value of 0.95 and a GC content of 52.04%. Since CAI values greater than 0.8 are considered to be good for expression in the target organism^64^, and since it is reported that a GC content between 30 and 70% is required for better expression^61^, the results of this section are satisfactory.

## Materials and methods

### Protein sequence retrieval

The reference sequence of E6 (NP_041325.1) and E7 (NP_041326.1) proteins from HPV16 were retrieved in FASTA format from NCBI database (https://www.ncbi.nlm.nih.gov/).

### Identification and selection of T-cell epitopes

The NetCTL 1.2 server (http://www.cbs.dtu.dk/services/NetCTL/) was used to identify the CTL epitopes for the target proteins^65^. This server can predict CTL epitopes (9 mer), restricted to 12 MHC class I supertypes, including A1, A2, A3, A24, A26, B7, B8, B27, B39, B44, B58, and B62. A combination of three approaches proteasomal C-terminal cleavage, TAP transport efficiency, and MHC class-I binding affinity, is included in the prediction. TAP transport efficiency is evaluated using a weight matrix, while MHC-I binding and proteasoma C-terminal cleavage are predicted using artificial neural networks. In this study, the threshold value for epitope prediction was set at 0.75.

The NetMHCII 2.3 server (http://www.cbs.dtu.dk/services/NetMHCII/) was used to identify the HTL epitopes^66^. This server predicts the binding of the HTL epitopes (15 mer) to HLA-DR, HLA-DQ, HLA-DP, and mouse MHC class II alleles using artificial neural networks. In this study, the thresholds for strong and weak binders were set at 2% and 10%, respectively.

Due to the large number of epitopes, epitope screening for antigenicity, toxicity, and allergenicity is performed to select the best epitopes. The VaxiJen v2.0 server (http://www.ddg-pharmfac.net/vaxijen/VaxiJen/VaxiJen.html) was used to predict the antigenicity of the epitopes^67–69^. This server is capable of calculating the antigenicity of various microorganisms, such as bacteria, viruses, tumors, parasites, and fungi. The accuracy of the prediction by the VaxiJen v2.0 server is between 70 and 89%. In this analysis, the virus was selected as the target organism, and the antigenicity threshold was set at 0.4. Moreover, the ToxinPred server was used to predict epitope toxicity^70^. In this study, an SVM-based method (Swiss-Prot) was selected to predict toxicity. In addition, the AllerTOP v. 2.0 server (https://www.ddg-pharmfac.net/AllerTOP/method.html) was used to evaluate the allergenicity of the epitopes^71^. The strategy utilized in this server is based on the auto cross covariance (ACC) change of amino acid sequences into standard vectors of identical length^72^. It is important to note that not all HTL epitopes have the ability to induce the production of cytokines, and if produced, the cytokines produced by each may be different. Moreover, IL4pred (https://webs.iiitd.edu.in/raghava/il4pred/design.php) and IFNepitope (https://webs.iiitd.edu.in/raghava/ifnepitope/design.php) were used to predict IL-4 and IFN-γ inducing HTL epitopes, respectively. The SVM-based model and a threshold of 0.2 were selected to predict IL-4 inducing HTL epitopes^73^, and an SVM-based and IFN-gamma versus other cytokine models were selected to predict IFN-γ inducing HTL epitopes^74^.

### Construction of the multi-epitope vaccine construct

Epitopes selected from the previous step were used to construct a multi-epitope vaccine. The HTL epitopes were linked using GPGPG linkers, whereas AAY linkers were used for the CTL epitopes. Linkers increase the representation and proper separation of the epitopes^28^. Moreover, glycine-rich linkers, such as GPGPG, also help improve solubility. Furthermore, the 50S ribosomal protein L7/L12 (Locus RL7_MYCTU) with accession no. P9WHE3 was selected as an adjuvant to enhance the immunogenicity of the vaccine candidate, and its amino acid sequence was attached by an EAAAK linker to the N-terminal of the chimeric sequences.

### Evaluation of the antigenicity, allergenicity, and physicochemical properties of the vaccine

The assessment of antigenicity is an essential step in the process of designing vaccines. Two servers, VaxiJen v2.0 and ANTIGENpro, were used to predict the antigenic behavior of the final vaccine construct. ANTIGENpro (http://scratch.proteomics.ics.uci.edu/) estimates protein antigenicity using five machine learning algorithms and multiple representations of the initial sequence^75^. In order to ensure that the vaccine was not allergenic, AllerTOP v. 2.0 was used to predict the allergenicity of the vaccine. In this study, we used the Expasy ProtParam server (https://web.expasy.org/protparam/) to predict various physicochemical parameters of the multi-epitope vaccine, including amino acid composition, theoretical pI, molecular weight, instability index, half-life, aliphatic index, and grand average of hydropathicity (GRAVY)^59^.

### Prediction of the secondary structure

We used the Prabi server (https://npsa-prabi.ibcp.fr/cgi-bin/npsa_automat.pl?page=/NPSA/npsa_gor4.html) to predict the percentage of secondary structure elements in the vaccine construct. GOR IV is the prediction method used on this server, which has a mean accuracy of 64.4%^76^.

### Tertiary structure modeling, refinement, and validation of the multi-epitope vaccine

The I-TASSER server (https://zhanglab.ccmb.med.umich.edu/I-TASSER/) was used to predict the 3D model of the multi-epitope vaccine. This server generates three-dimensional structures from the amino acid sequence by reassembling the excised parts from the threading templates, and it calculates the C-score to evaluate the accuracy of the predicted models^22,77,78^. The selected model was refined using the 3Drefine server (http://sysbio.rnet.missouri.edu/3Drefine/) to improve its structural quality. The algorithm used in the 3Drefine server includes a two-step process (1) the optimization of the hydrogen bonding network, and (2) the minimization of atomic energy by integrating physics into the force field^79–81^. Model validation was performed using the ProSA-web server (https://prosa.services.came.sbg.ac.at/prosa.php) and the SAVES v6.0 server (https://saves.mbi.ucla.edu/). The ProSA-web server computes the overall quality Z-score for the protein structure. If the Z-score is outside the characteristic range of the native proteins, it indicates that there may be errors in the protein structure^82,83^. The PROCHECK tool of the SAVES v6.0 server evaluates the stereochemical quality of a protein structure by checking the geometry of the residues and the overall structural geometry^84,85^.

### Prediction of the B-cell epitopes

The most important elements in the immune system are B lymphocytes, which are responsible for antibody secretion, thus promoting long-term immunity ^28^. The prediction of linear B-cell epitopes was performed using the BCPREDS (B-cell epitope prediction server) (http://ailab-projects1.ist.psu.edu:8080/bcpred/predict.html). This server uses a subsequence kernel-based SVM classifier with an accuracy of 74.57% to predict linear B-cell epitopes^86–88^. Furthermore, the Ellipro server (http://tools.iedb.org/ellipro/) was used for the prediction of discontinuous B-cell epitopes. The ElliPro server uses residue clustering algorithms along with the Tornton’s method for predicting discontinuous B-cell epitopes. The server assigns a score to each of the predicted epitopes, which is defined as a PI (protrusion index) value^89^.

### Molecular docking

Molecular docking is one of the computational methods used to evaluate the interaction between two molecules and find the best orientation of a ligand in a complex. The ClusPro 2.0 server (https://cluspro.org/login.php) was used to evaluate the interaction between the vaccine construct and TLR4 (PDB ID: 4G8A)^90–93^. The refined model of the vaccine construct, as the ligand, and TLR4, as the receptor, were submitted to the server. The LigPlot program was used to illustrate the bonds that formed between the residues of the vaccine construct and TLR4 in the docked complex^24^.

### MD simulation

The MD simulation was performed using the GROMACS 2019.6 software. Using Newton’s laws of atomic and molecular motion, the software predicts the behavior of ligands and receptors over a specific period of time^94–96^. The ff99SB force field was used to prepare the input structure. The surface charge of the structure was then neutralized using sodium and chloride ions. The gmx solvate software was also used to insert the protein into a layer of TIP3P water molecules with a thickness of 10 angstroms. To eliminate van der Waals interactions and hydrogen bonds forming between the water and the complex molecules, the energy of the structures was minimized using the steepest descent method. Afterward, the system temperature was steadily increased from 0 to 300 K in a constant volume for 200 ps, and the system was then equilibrated at constant pressure. Finally, the root-mean-square deviation (RMSD) and root-mean-square fluctuation (RMSF) of the ligand and the receptor were calculated over a 40-ns timeframe.

### Codon optimization and in silico cloning of the final vaccine construct

The Java Codon Adaptation Tool (JCat) (http://www.jcat.de/) was used for the back translation and codon optimization of the final vaccine construct^97^. The protein sequence of the vaccine was submitted to the JCat, and *E. coli* (K12 strain) was selected as the host organism to express the vaccine construct. This server calculates two parameters, the codon adaptive index (CAI) and the GC content, which are important for the evaluation of protein expression levels. The sequence of restriction sites for XhoI and BamHI restriction enzymes were introduced at the 5′ and 3′ ends of the vaccine construct, respectively, and the vaccine sequence was then cloned into the pET28 (+) vector using the SnapGene software (https://www.snapgene.com/free-trial/).

## Conclusions

Cervical cancer, caused by HPV, has affected the health of millions of people worldwide. There is currently no effective therapeutic vaccine available to treat HPV infections. In this study, we have attempted to design a multi-epitope vaccine against cervical cancer using reverse vaccinology. CTL and HTL epitopes from the E6 and E7 proteins of HPV16 were identified Antigenicity, toxicity, and allergenicity of the predicted epitopes were assessed, and the best epitopes were merged using appropriate linkers and adjuvant. The designed vaccine was found to be both antigenic and non-allergenic, and its physicochemical properties were acceptable. Molecular docking was also performed to check the binding affinity of the vaccine construct with TLR-4 in the vaccine-TLR4 complex. The stability of the vaccine candidate was confirmed by MD simulation. At last, the expression and translation efficiency of the multi-epitope vaccine was evaluated. Although the findings of this study were very impressive, they must be validated in the wet lab and animal models.
